# Comparing Characteristics of Sporadic and Outbreak-Associated Foodborne Illnesses, United States, 2004–2011

**DOI:** 10.3201/eid2207.150833

**Published:** 2016-07

**Authors:** Eric D. Ebel, Michael S. Williams, Dana Cole, Curtis C. Travis, Karl C. Klontz, Neal J. Golden, Robert M. Hoekstra

**Affiliations:** US Department of Agriculture District of Columbia, Washington, DC, USA (E.D. Ebel, M.S. Williams, N.J. Golden);; Centers for Disease Control and Prevention, Atlanta, Georgia, USA (D. Cole, R.M. Hoekstra);; Leidos Incorporated, Reston, Virginia, USA (C.C. Travis);; Food and Drug Administration, College Park, Maryland, USA (K.C. Klontz)

**Keywords:** disease outbreaks, foodborne diseases, Salmonella, Listeria, Campylobacter, Escherichia coli O157, sporadic, FoodNet, Foodborne Diseases Active Surveillance Network, enteric infections, bacteria

## Abstract

Outbreak data have been used to estimate the proportion of illnesses attributable to different foods. Applying outbreak-based attribution estimates to nonoutbreak foodborne illnesses requires an assumption of similar exposure pathways for outbreak and sporadic illnesses. This assumption cannot be tested, but other comparisons can assess its veracity. Our study compares demographic, clinical, temporal, and geographic characteristics of outbreak and sporadic illnesses from *Campylobacter*, *Escherichia coli* O157, *Listeria*, and *Salmonella* bacteria ascertained by the Foodborne Diseases Active Surveillance Network (FoodNet). Differences among FoodNet sites in outbreak and sporadic illnesses might reflect differences in surveillance practices. For *Campylobacter*, *Listeria,* and *Escherichia coli* O157, outbreak and sporadic illnesses are similar for severity, sex, and age. For *Salmonella*, outbreak and sporadic illnesses are similar for severity and sex. Nevertheless, the percentage of outbreak illnesses in the youngest age category was lower. Therefore, we do not reject the assumption that outbreak and sporadic illnesses are similar.

## Abstract 2

Our findings do not warrant rejecting the hypothesis that outbreak and sporadic illnesses are similar.

Comparing Sporadic and Outbreak Foodborne Illness

A previous study used outbreak data to determine the relative contributions of 17 different food commodities to the annual prevalence of foodborne illness in the United States (*1*). That work assumed that the exposure pathways of foodborne outbreak illnesses were representative of those pathways for all foodborne illnesses, including outbreak-associated and sporadic (nonoutbreak) illnesses. However, this assumption cannot be tested directly because the food sources of sporadic illnesses typically are unknowable. In fact, despite the availability of multiple cases and controls that might enable examination of the likelihood of illness for different foods consumed, the food sources of outbreaks are identified in only about one half of all foodborne disease outbreaks investigated (*2*).

In lieu of a direct comparison of exposure pathways between outbreak and sporadic foodborne illnesses, we compare selected demographic, clinical, temporal, and geographic characteristics of outbreak and sporadic cases of illness caused by *Campylobacter*, *Escherichia coli* O157, *Listeria*, and *Salmonella* bacteria by using data from the Foodborne Diseases Active Surveillance Network (FoodNet) for 2004–2011. Such an analysis is limited but still useful. Although similarities between outbreak and sporadic cases in terms of disease characteristics would not imply that these cases have identical food exposures, notable differences in disease characteristics might indicate differences in food exposures.

## Methods

Data submitted to the Centers for Disease Control and Prevention (CDC) by public health personnel from each FoodNet site indicate whether a case of foodborne illness is an outbreak or nonoutbreak (sporadic) case. We aimed to determine whether differences exist in terms of 6 selected characteristics of outbreak cases of laboratory-confirmed *Campylobacter*, *E. coli* O157, *Listeria*, and *Salmonella* infection reported in FoodNet (*3*) during 2004–2011. The 6 characteristics examined were as follows: 1) the FoodNet site reporting the case; 2) the year in which a case occurred; 3) the season in which a case occurred; 4) the age of patient (generally, the difference between submission date and reported date of birth); 5) the sex of the patient; and 6) the hospitalization status of the patient (i.e., whether the patient was hospitalized within 7 days of specimen collection).

Since 2004, the FoodNet surveillance catchment area has been stable. The FoodNet sites were Connecticut, Georgia, Maryland, Minnesota, New Mexico, Oregon, Tennessee, and selected counties in California, Colorado, and New York. To ensure sufficient data, we determined quintiles for season and age groups. Because the data distributions differed between the pathogens, these quintiles were determined for each pathogen separately. Sex and hospitalization status were binary variables.

Other variables of potential interest, such as source of specimen (e.g., stool, blood, or urine), race, ethnicity, and international travel, were not included in the analysis because there were relatively high percentages of missing observations for some pathogens and because percentages were highly variable over time and across other variables in the analysis, possibly introducing an unknown amount of surveillance bias and limiting interpretation of results. For example, the fraction of cases for which information on international travel by the patient was missing ranged from 6% for *E. coli* O157 to 44% for *Campylobacter*. Similarly, the fraction of cases for which information on race was missing ranged from 7% for *E. coli* O157 to 26% for *Campylobacter*. Our summary descriptions and final models are based on the set of FoodNet case reports for which all 6 variables are complete. Missing values for certain variables are described in the Technical Appendix.

To complete the analysis of these characteristics, we used a 2-step approach for each of the 4 pathogens examined. First, we conducted random forest and boosted tree analyses (*4*,*5*) to gauge the relative importance of the 6 characteristics in distinguishing between outbreak and sporadic cases. Random forest analysis is a data classification algorithm that seeks the best combination of factors to explain an outcome variable (i.e., outbreak or sporadic case). Boosted tree analysis pertains to the use of regression techniques (e.g., mean square errors) for measuring the fit of the trees to the data. We created random collections of classification trees and averaged those trees by a measure of how well each tree fit the data.

For each pathogen, we trained random forest models on ≈85% of the data; we used the remaining ≈15% of the data to validate the model’s classifications of outbreak and sporadic cases. We used the G^2^ statistic (a modified Wilk’s statistic) to identify more and less important factors (*6*). In a stepwise fashion, we removed the least important factors to determine if model misclassification of outbreak status improved for the training dataset or the validation dataset. We stopped the model simplification whenever removal of a factor caused misclassification to worsen. Factors that were not eliminated were carried on to the next step.

The second step of the analysis was logistic regression modeling. We used stepwise model building routines (*7*) to examine all main effects and interactions among the factor levels (i.e., model parameters) explaining the fraction of cases that are outbreak-associated cases (i.e., 

 where *p* is the probability of a case being an outbreak case and *X* is a matrix of the data with the number of rows equal to the number of cases and the number of columns equal to the total levels of explanatory variables considered). As a model identification guide, we used forward selection procedures and minimum Bayesian information criterion scoring (BIC) (*8*). BIC is a preferred selection criterion because it penalizes the inclusion of additional parameters more strongly than alternative statistics (e.g., Akaike information criteria) (*8*,*9*).

We selected the logistic regression models with the lowest BIC scores as the best models. We used visual assessments of the residuals and interactions to assess the adequacy of the methods and models.

## Results

During the study period (2004–2011), <1% of *Campylobacter* infections reported in FoodNet were outbreak cases, but ≈20% of *E. coli* O157 infections were outbreak cases. Outbreak cases represented ≈5% of *Listeria* and *Salmonella* infections (Table 1).

**Table 1:** Number of outbreak cases versus sporadic cases and outbreak fraction, FoodNet data, United States, 2004–2011*

Pathogen	Outbreak cases	Sporadic cases	Outbreak fraction, %
*Campylobacter*	195	42,744	0.5
*Escherichia coli* O157	730	3,117	19.0
*Listeria*	56	1,024	5.2
*Salmonella*	3,161	50,690	5.9


*Representing 101,717 reports with complete data for all study variables out of 110,157 total reports. FoodNet, Foodborne Diseases Active Surveillance Network.

**Figure 1:**
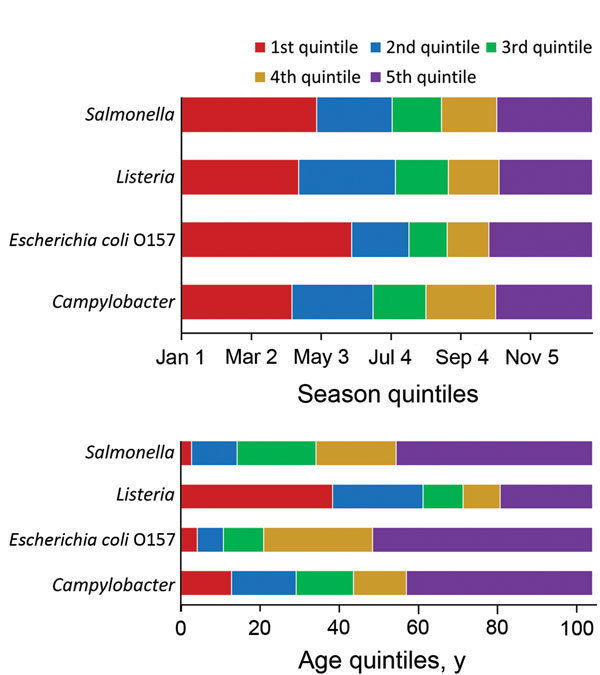
Quintile categorization of season and age for persons with foodborne illness included in the analysis of Foodborne Diseases Active Surveillance Network (FoodNet) data, United States, 2004–2011.

Seasonal quintiles were similar across pathogens except for *E. coli* O157; the first season was longer compared with the other pathogens, extending from January through the end of May (Figure 1). Age quintiles, however, differed substantially across pathogens. For example, to capture 20% of the data for *Listeria*, the first quintile was defined as cases in patients who were 0–38 years of age. In contrast, the first quintile for *Salmonella* only extended to patients 3 years of age. For *Listeria*, the relatively narrow quintile range for persons 60–80 years of age reflects the larger number of older persons among these cases. For the binary variables (sex and hospitalization), the frequency of male patients was ≈50% among all FoodNet cases for the 4 pathogens, and the percentages hospitalized for *Campylobacter*, *E. coli* O157, *Listeria,* and *Salmonella* infections were 16%, 44%, 93%, and 29%, respectively.

A descriptive treatment of the data shows that the frequency of outbreak cases among all FoodNet cases varied more for FoodNet site, year, patient age, and season than for sex and hospitalization status for each pathogen (Table 2). Compared with the other pathogens, *Listeria* exhibited substantial frequency ranges for some characteristics. For example, the percentage of *Listeria* cases that were outbreak versus sporadic cases per year varied from 0% versus 100% during 2007–2009 to 30.6% versus 69.4% in 2011. Variability was difficult to determine for *Campylobacter* because of the low frequency of outbreak-associated cases.

**Table 2:** Percentage of cases and total number of cases identified as outbreak-associated, by target pathogen and selected characteristics, FoodNet data, United States, 2004–2011*

Characteristic	% Outbreak cases (no. total observations)			
	*Campylobacter*	*Escherichia coli* O157	*Listeria*	*Salmonella*
FoodNet site				
California	0.1 (5,552)	1.5 (264)	1.7 (115)	3.0 (3,764)
Colorado	1.0 (3,391)	38.9 (319)	33.3 (72)	8.6 (2,491)
Connecticut	0.0 (3,689)	17.0 (277)	0.0 (148)	6.5 (3,335)
Georgia	0.2 (4,815)	8.4 (261)	0.0 (176)	2.6 (17,215)
Maryland	0.6 (2,920)	13.0 (200)	0.7 (140)	4.3 (6,020)
Minnesota	0.5 (7,308)	20.1 (1,078)	3.4 (58)	10.3 (5,379)
New Mexico	0.8 (2,640)	10.9 (92)	34.9 (43)	9.3 (2,497)
New York	0.4 (4,277)	22.9 (393)	3.7 (136)	8.2 (3,772)
Oregon	0.9 (5,147)	25.5 (545)	8.1 (86)	20.5 (3,067)
Tennessee	0.4 (3,200)	12.2 (418)	0.0 (106)	3.0 (6,311)
Year				
2004	0.2 (4,770)	9.0 (387)	0.8 (119)	6.0 (5,676)
2005	0.7 (5,009)	22.7 (467)	1.5 (136)	4.3 (5,982)
2006	0.7 (4,903)	15.9 (567)	4.4 (137)	7.6 (5,901)
2007	0.1 (5,377)	17.8 (546)	0.0 (122)	6.2 (6,540)
2008	0.6 (5,291)	25.8 (516)	0.0 (134)	7.9 (7,214)
2009	0.3 (5,546)	26.4 (458)	0.0 (157)	5.5 (6,844)
2010	0.4 (5,852)	21.1 (445)	2.3 (131)	5.2 (8,073)
2011	0.6 (6,191)	11.7 (461)	30.6 (144)	4.6 (7,621)
Age quintile				
1	0.7 (8,563)	20.6 (766)	2.3 (214)	2.2 (10,838)
2	0.7 (8,614)	18.1 (768)	4.6 (216)	4.4 (10,666)
3	0.3 (8,428)	19.3 (774)	5.1 (216)	9.2 (10,686)
4	0.3 (8,634)	19.6 (765)	5.5 (218)	7.7 (10,758)
5	0.3 (8,700)	17.3 (774)	8.3 (216)	6.0 (10,903)
Season quintile				
1	0.4 (8,552)	18.6 (774)	2.3 (218)	6.9 (10,962)
2	0.4 (8,761)	19.8 (773)	0.9 (215)	7.6 (10,804)
3	0.6 (8,545)	18.8 (775)	4.1 (218)	5.8 (10,773)
4	0.6 (8,666)	20.1 (770)	16.1 (217)	4.3 (10,671)
5	0.2 (8,415)	17.5 (755)	2.4 (212)	4.7 (10,641)
Sex				
F	0.4 (19,317)	19.4 (2,030)	6.4 (577)	6.1 (28,102)
M	0.4 (23,622)	18.4 (1,817)	3.8 (503)	5.4 (25,749)
Hospitalized				
No	0.5 (35,962)	20.1 (2,145)	4.1 (74)	6.3 (38,321)
Yes	0.3 (6,977)	17.5 (1,702)	5.3 (1,006)	4.8 (15,530)

*Age of persons with cases and season of specimen submission are classified by quintile of reported age and quintile of the day of year of the specimen submission date. FoodNet, Foodborne Diseases Active Surveillance Network.

In general, FoodNet sites in Georgia and California had smaller percentages of outbreak cases, whereas Oregon and Colorado had larger percentages. California had small outbreak case percentages for *Campylobacter* (0.1%) and *E. coli* O157 (1.5%), whereas Georgia had the smallest percentage among all sites for *Listeria* (0.0%) and *Salmonella* (2.6%). Colorado had the largest outbreak case percentage among all sites for *Campylobacter* (1.0%) and *E. coli* O157 (38.9%), whereas Oregon and New Mexico had the largest percentages for *Salmonella* (20.5%) and *Listeria* (34.9%), respectively.

For each pathogen’s random forest analysis, the G^2^ statistic was smallest for the binary variables (sex and hospitalization). Furthermore, misclassification errors for the training and validation datasets were not substantively changed whether the analysis included all 6 factors or excluded sex and hospitalization status. Consequently, sex and hospitalization status were not important for classifying outbreak and sporadic cases for any of the pathogens, and these factors were excluded from the logistic modeling step.

**Figure 2:**
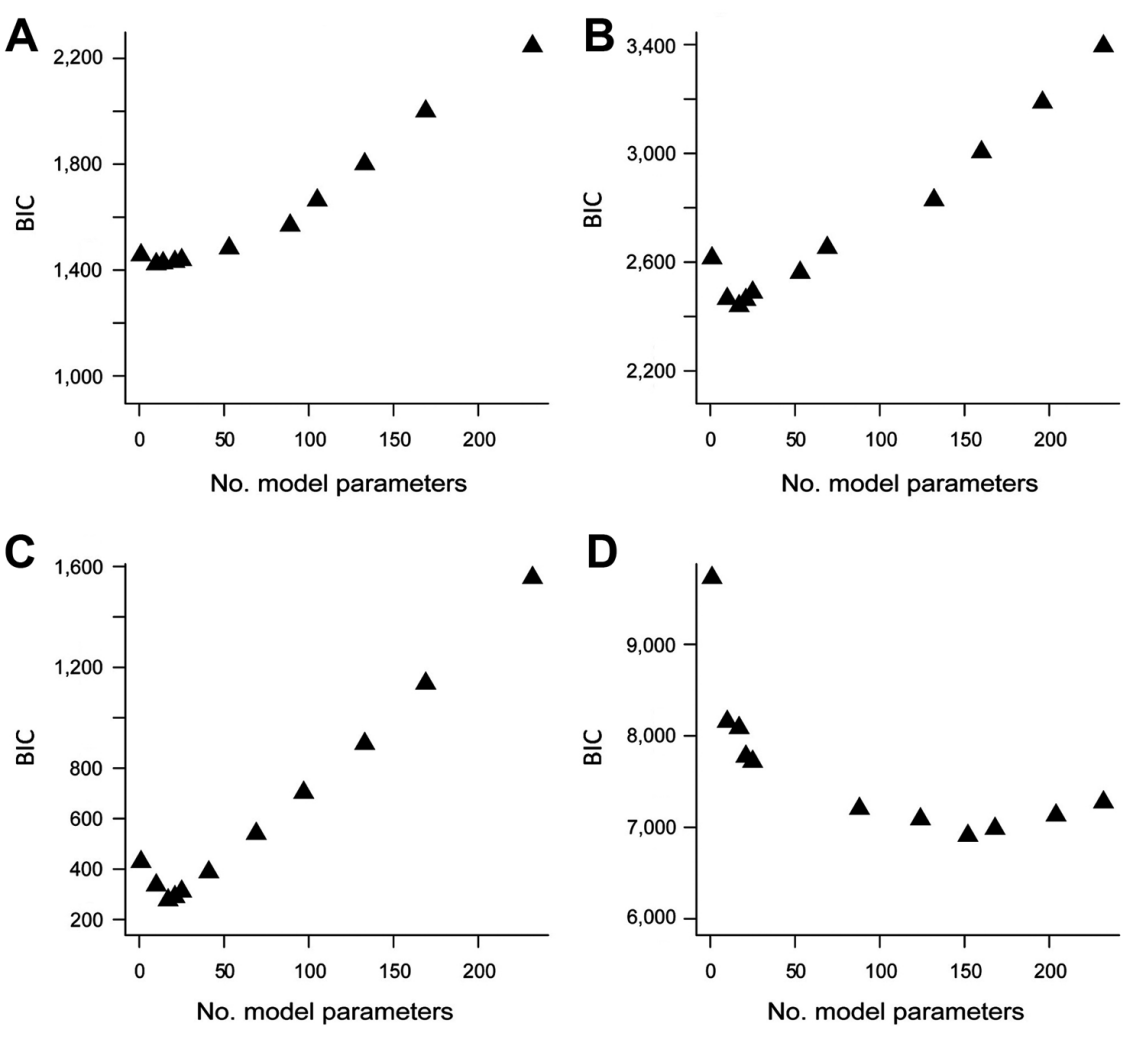
Patterns of the Bayesian information criterion (BIC) statistic as a function of the number of model parameters are shown for the four pathogens included in the analysis of Foodborne Diseases Active Surveillance Network (FoodNet) data, United States, 2004–2011. A) *Campylobacter*; B) *Escherichia coli* O157; C) *Listeria*; D) *Salmonella*. The BIC decreases to a minimum value and then increases as model complexity (as measured by the number of model parameters) increases.

**Figure 3:**
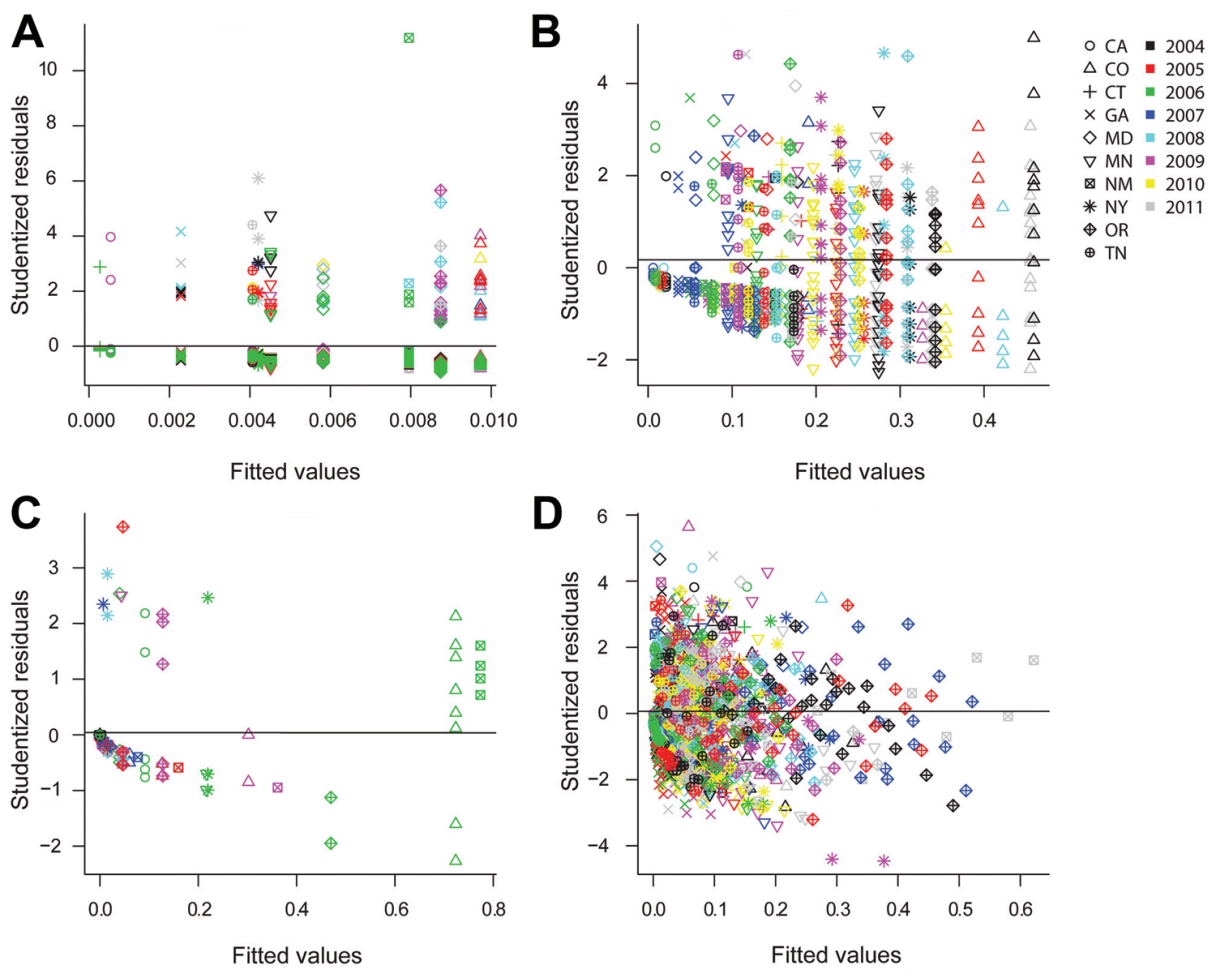
Residual plots relative to fitted estimates of outbreak-associated case frequency for the best-fitting models used in the analysis of Foodborne Diseases Active Surveillance Network (FoodNet) data, United States, 2004–2011. A) *Campylobacter*; B) *Escherichia coli* O157; C) *Listeria*; D) *Salmonella*. Generally, all 4 pathogen models demonstrate reasonable fit because the studentized residuals ([observed frequency – predicted frequency of outbreak-associated cases]/SE of predicted frequency) are mostly within 3 SD of the predicted mean frequency of outbreak-associated cases. The state variable is the only factor in the *Campylobacter* model, whereas year is included in the *E. coli* O157 and *Listeria* models. The *Salmonella* model includes state, year, season, age, and interaction terms.

Plots of the BIC statistic for increasingly complex models illustrate that its value decreases to a minimum and then increases for more complicated models (Figure 2). For *Campylobacter*, the minimum BIC corresponds to a model containing just the FoodNet site parameters. For *E. coli* O157 and *Listeria*, the minimum BIC corresponds to a model with 16 parameters (9 for FoodNet site and 7 for year, with 1 reference value for each factor included in the intercept term). For *Salmonella*, the minimum BIC corresponds to a model with 152 parameters that includes all 4 factors (24 parameters plus the reference intercept), the FoodNet site by year interactions (63 parameters), the year by season interactions (28 parameters), and the FoodNet site by season interactions (36 parameters). Residual plots of the best-fitting models demonstrate reasonable fit to the data (Figure 3). These plots illustrate that the studentized residuals ([observed frequency – predicted frequency of outbreak-associated cases]/SE of predicted frequency) generally cluster within 3 SD of the mean.

**Figure 4:**
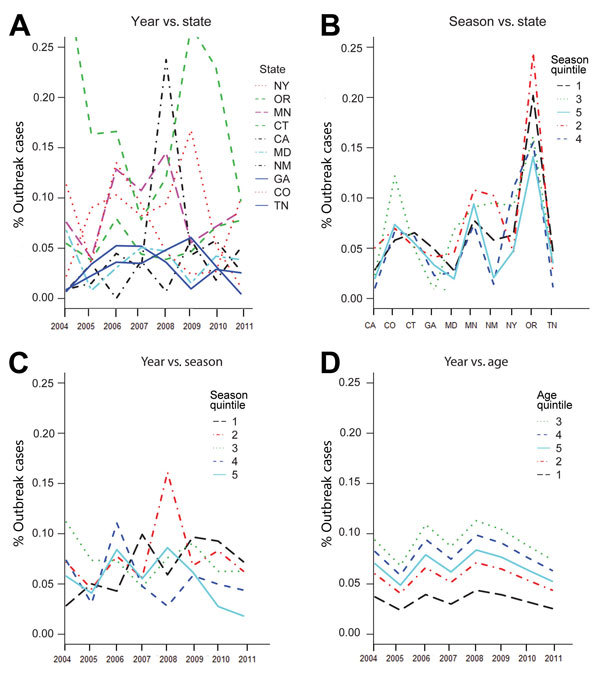
Interaction plots from the best-fitting *Salmonella* logistic regression model used in the analysis of Foodborne Diseases Active Surveillance Network (FoodNet) data, United States, 2004–2011. A) Year versus state; B) season versus state; C) year versus season; D) year versus age. The y-axis is the proportion of outbreak-associated cases. Crossing lines indicate interactions between 2 factors for the proportion of outbreak-associated case.

Interaction plots from the best-fitting *Salmonella* model (Figure 4) illustrate the complex relationships between some model factors. For example, interaction plots demonstrated that, for some FoodNet sites (e.g., Oregon, California, and Minnesota), the estimated proportion of outbreak-associated cases can change substantially across years. Moreover, the directions of changes are inconsistent across the sites. For example, the peaks and troughs of Oregon’s proportions across years are nearly the opposite of Minnesota’s pattern. Likewise, the *Salmonella* interaction plots demonstrated interactions between the seasonal quintile and both the surveillance year and the FoodNet site. In contrast, the patterns for the age quintiles are consistent across surveillance years. Nevertheless, the first age quintile (0–3 years of age) has a markedly lower proportion of outbreak-associated cases relative to the other age quintiles. This underrepresentation of outbreak-associated cases among the youngest age quintile drives the significance of the age parameter in the logistic regression model.

## Discussion

If foodborne illness source attribution estimates are to be effectively used for food safety decision making and monitoring success of interventions, the data used to generate them must be collected in a systematic fashion over time. Foodborne outbreak surveillance data have been systematically collected since 1973 and provide direct links between human illnesses and food sources. Although other methods of source attribution (e.g., case–control studies) can provide relevant estimates for different target populations, these estimates are potentially expensive, logistically complex, and not routinely conducted in the United States. Moreover, estimated attributable fractions are based on associations between illnesses and exposures, not proof of causality. The possibility that attribution estimates from outbreaks might not be reliably generalized to the bulk of estimated foodborne illnesses is recognized (*1*). Nevertheless, we cannot assess directly the validity of outbreak-based attribution estimates for application to the broader population of foodborne illnesses. Consequently, this study assessed similarities and differences between outbreak and sporadic cases across various case characteristics. If the examined characteristics of outbreak and sporadic cases are different for these data, then the assumption of similar exposure pathways is less plausible. FoodNet is particularly well-suited for this analysis, because it is the only US foodborne disease surveillance system that actively ascertains laboratory-confirmed human infections and distinguishes those cases that are associated with detected outbreaks.

In our analysis, the probability of a case being outbreak-associated varied significantly across the FoodNet surveillance sites for all 4 pathogens studied. Uncertainty exists for the causes of variability in the number of ascertained cases across FoodNet sites (*10*) and the number of outbreaks detected and reported across states (*2*,*11*,*12*). Previous research has demonstrated that differences in specimen collection and testing and outbreak surveillance and reporting practices, contribute to differences among states, and differences in funding or resource allocation have been hypothesized to be influential factors (*2*,*10*–*12*). We assume these sources of variability among sites are most influenced by differences in surveillance and do not suggest underlying differences in the sources of sporadic and outbreak illnesses.

The probability of a case being outbreak-associated also varied significantly with the surveillance year for *E. coli* O157, *Listeria,* and *Salmonella*. In addition, the season of specimen submission was a significant factor in the *Salmonella* model. In a study by Painter et al. (*1*), source attribution was estimated by aggregating multiple years of outbreak data and applying those to national annual burden of illness estimates (*13*). Gould et al. (*2*) similarly aggregated outbreak data for estimating source attribution. One justification for aggregating outbreak evidence across years (and seasons) is the need to capture more information than is available from a single year (or season). The significant association between the probability of an outbreak case and year (and state and season) suggests that aggregation of outbreak data across time and space might be appropriate to avoid biases introduced by significant local effects. Outbreak and sporadic cases might be dissimilar across periods of ≈1 year but more similar when multiple years are compared. For example, the fraction of outbreak-associated cases in the FoodNet *Salmonella* data are 5.7% for 2004–2007 and 5.8% for 2008–2011, despite year-to-year fluctuations ranging from 4.3% to 7.9% (Table 2).

Our analysis found no evidence that laboratory-confirmed outbreak and sporadic cases are dissimilar with respect to the sex or hospitalization status of patients. In particular, the data for *Salmonella* and *E. coli* O157 include substantial numbers of cases for comparisons of these factors. Therefore, the conclusion from the random forest analysis regarding these pathogens lends support to the same conclusion for the other 2 pathogens. Otherwise, the small number of outbreak-associated cases for *Campylobacter* and the generally small number of *Listeria* cases provides limited statistical power to detect real differences.

In the case of *Salmonella*, this analysis found that the percentage of outbreak-associated cases varied significantly by age cohort. In fact, the youngest age quintile (0–3 years of age) had the smallest proportion of outbreak-associated cases. Given this result, applying source attribution estimates derived from foodborne outbreak data to the youngest age strata of *Salmonella* sporadic cases might not be prudent. Because FoodNet epidemiologists cannot confirm the exposure pathway that resulted in FoodNet-captured illnesses, we cannot determine whether the lower frequency of outbreak-associated cases among the youngest cohorts of *Salmonella* patients reflects some fundamental difference in the distribution of exposure pathways, a difference in outbreak-associated case detection methods, or both.

The analytical methods we used rely on some assumptions. The initial random forest analysis was completed because this technique demands few assumptions with respect to missing observations and factor interactions (*14*). Nevertheless, this technique was only used to eliminate those factors that had no evident association with outbreak status.

The logistic regression modeling we performed relies on a binomial process assumption for the frequency of outbreak cases among all FoodNet cases. Although this analysis assumes that all outbreak cases are unrelated to each other, detailed data about the specific outbreak for each outbreak case is not readily available and some outbreak cases might have stemmed from the same outbreak. Related outbreak cases might co-vary with respect to the factors we studied in violation of the binomial process assumption of independent trials. To address this possibility, we considered censoring outbreak cases in this analysis, but an unknown number of sporadic cases probably were also related to detected and undetected outbreaks.

This study also assumes that the probability of specimen collection and laboratory submission among ill persons is the same for outbreak and sporadic cases. Nevertheless, public awareness of an outbreak might increase healthcare-seeking behavior and submission of diagnostic samples by healthcare providers. In addition, during some outbreak investigations, investigators conduct active case-finding and collect additional laboratory specimens from persons reporting foodborne illness (*11*,*15*), resulting in laboratory-confirmed infections being identified in persons who had not sought healthcare. As a result, outbreak cases might be oversampled compared with sporadic infections.

Inherent dependencies among outbreak cases, combined with oversampling, might contribute to an increased strength of association between the proportion of outbreak-associated cases and the factors studied here. In addition to performing better than alternative criteria when the objective of modeling is to find the actual model, BIC penalizes the addition of parameters in models more harshly (*16*). We believe that this harsher assessment of factors reduces the likelihood of spurious associations.

Some of the persons with foodborne infections that were captured by FoodNet traveled internationally before their reported specimen collection date, and some of these persons probably became infected because of exposures that occurred outside the United States. The likelihood of their illness being associated with a disease outbreak might in turn be different from that of non-travelers. We were not able to exclude international travelers or adjust for this case characteristic because, except for cases of *E. coli* O157 infection, travel history information was missing for >20% of cases. Thus, our study population is not restricted to persons with infection caused by domestic exposures. Nevertheless, international travel was reported for <10% of cases for all pathogens except *Campylobacter*. Among *Campylobacter* infection cases in persons who reported a travel history, 18% involved international travel before illness onset; however, the small number of outbreak-associated cases is probably the primary limitation of the *Campylobacter* analyses.

We conclude that the characteristics of outbreak and sporadic cases captured by FoodNet vary for all 4 pathogens examined. Nevertheless, with the exception of season and age of patient for *Salmonella* cases, the differences between outbreak and sporadic cases pertain to factors that are probably associated with the inherent variability among complex surveillance systems. Our finding with respect to age differences for *Salmonella* outbreak and sporadic case-patients suggests that applying outbreak-based source attribution estimates to the youngest case-patients might be inappropriate. Otherwise, because our analysis generally finds that outbreak and sporadic illnesses have similar case characteristics, our impression is that this study does not refute the plausibility of outbreak-based source attribution methods demonstrated in Painter et al. (*1*).

Our study was limited to cases that were laboratory-confirmed. Consequently, our conclusions are based on the assumption that persons with foodborne illness who did not seek healthcare or did not have a specimen submitted for laboratory testing, are similar to those whose cases were included in our study. Nonetheless, source attribution methods will continue to evolve and will probably include data from multiple study populations. Recently, blending of outbreak-based and case-control source attribution estimates was evaluated (*15*). In the future, the type of analysis reported here could be used to examine more detailed case characteristics of illnesses transmitted commonly by food for similarities and differences between outbreak and sporadic cases. Currently, these types of data are not captured routinely in the US surveillance systems.

Technical Appendix Description of missing values for certain variables included in the analysis of Foodborne Diseases Active Surveillance Network (FoodNet) data, United States, 2004–2011.

## References

[1] Painter JA, Hoekstra RM, Ayers T, Tauxe RV, Braden CR, Angulo FJ, Attribution of foodborne illnesses, hospitalizations, and deaths to food commodities, United States, 1998–2008. Emerg Infect Dis. 2013;19:407–15 . 10.3201/eid1903.111866 23622497 PMC3647642

[2] Gould LH, Walsh KA, Vieira AR, Herman K, Williams IT, Hall AJ, Surveillance for foodborne disease outbreaks—United States, 1998–2008. MMWR Surveill Summ. 2013;62:1–34. 23804024

[3] Scallan E, Mahon BE. Foodborne Diseases Active Surveillance Network (FoodNet) in 2012: a foundation for food safety in the United States. Clin Infect Dis. 2012;54(Suppl 5):S381–4 . 10.1093/cid/cis257 22572657 PMC3348949

[4] De’ath G. Boosted trees for ecological modeling and prediction. Ecology. 2007;88:243–51 . 10.1890/0012-9658(2007)88[243:BTFEMA]2.0.CO;2 17489472

[5] Friedman J, Hastie T, Tibshirani R. Additive logistic regression: a statistical view of boosting (with discussion and a rejoinder by the authors). Ann Stat. 2000;28:337–407 . 10.1214/aos/1016218223

[6] Hélie S. Understanding statistical power using noncentral probability distributions: chi-squared, G-squared, and ANOVA. Tutor Quant Methods Psychol. 2007;3:63–9.

[7] R Development Core Team. R: a language and environment for statistical computing [cited 2015 Dec 12]. http://www.R-project.org

[8] Schwarz G. Estimating the dimension of a model. Ann Stat. 1978;6:461–4 . 10.1214/aos/1176344136

[9] Akaike H. Information theory and an extension of the maximum likelihood principle. In: Selected papers of Hirotugu Akaike. New York: Springer; 1998. p. 199–213.

[10] Ailes E, Scallan E, Berkelman RL, Kleinbaum DG, Tauxe RV, Moe CL. Do differences in risk factors, medical care seeking, or medical practices explain the geographic variation in campylobacteriosis in Foodborne Diseases Active Surveillance Network (FoodNet) sites? Clin Infect Dis. 2012;54(Suppl 5):S464–71 . 10.1093/cid/cis050 22572671

[11] Murphree R, Garman K, Phan Q, Everstine K, Gould LH, Jones TF. Characteristics of foodborne disease outbreak investigations conducted by Foodborne Diseases Active Surveillance Network (FoodNet) sites, 2003–2008. Clin Infect Dis. 2012;54(Suppl 5):S498–503 . 10.1093/cid/cis232 22572675

[12] Jones TF, Rosenberg L, Kubota K, Ingram LA. Variability among states in investigating foodborne disease outbreaks. Foodborne Pathog Dis. 2013;10:69–73 . 10.1089/fpd.2012.1243 23249418

[13] Scallan E, Hoekstra RM, Angulo FJ, Tauxe RV, Widdowson M-A, Roy SL, Foodborne illness acquired in the United States—major pathogens. Emerg Infect Dis. 2011;17:7–15 . 10.3201/eid1701.P11101 21192848 PMC3375761

[14] Breiman L. Random Forests. Mach Learn. 2001;45:5–32 . 10.1023/A:1010933404324

[15] Cole D, Griffin PM, Fullerton KE, Ayers T, Smith K, Ingram LA, Attributing sporadic and outbreak-associated infections to sources: blending epidemiological data. Epidemiol Infect. 2014;142:295–302 . 10.1017/S0950268813000915 23611460 PMC9151115

[16] Kuha J. AIC and BIC comparisons of assumptions and performance. Sociol Methods Res. 2004;33:188–229 . 10.1177/0049124103262065

