# Supplementary material for: Comparing Characteristics of Sporadic and Outbreak-Associated Foodborne Illnesses, United States, 2004–2011
Source: Emerg Infect Dis. 2016 Jul;22(7):1193–200. doi: 10.3201/eid2207.150833 (PMC4918141; doi:10.3201/eid2207.150833)
Supplement: Technical Appendix — Description of missing values for certain variables included in the analysis of Foodborne Diseases Active Surveillance Network (FoodNet) data, United States, 2004–2011. [file 15-0833-Techapp-s1.pdf]

Article DOI: http://dx.doi.org/10.3201/eid2207.150833

Comparing Characteristics of Sporadic and Outbreak-Associated Foodborne Illnesses, United States, 2004–2011

Technical Appendix

The frequency of missing values among the 6 selected characteristics was very low except for hospitalization status. We ran sensitivity analyses by pathogen for hospitalization status, randomly assigning status and separately forcing all missing values to “yes” and “no”. These analyses did not alter any conclusions from the study. Age and season quintiles were very similar across all pathogens. Because these were defined to maintain sample size with cross-classifications, the quintiles defined based on all data were used in the final analyses.

Technical Appendix Table 1. Percentage of cases of foodborne illness with missing values, by pathogen, Foodborne Diseases Active Surveillance Network (FoodNet) data, United States, 2004–2011

	% Cases with missing variables			
Characteristic	*Campylobacter*	*Escherichia coli* O157	*Listeria*	*Salmonella*
Outbreak status	—	—	—	0.002
Age	0.06	0.05	—	0.10
Sex	0.09	0.10	0.09	0.26
Hospitalization	10.62	1.30	0.28	5.43

*There were no missing values for FoodNet reporting site or specimen submission date (and thus year and season). One record was missing outbreak status. This table represents 110,157 total reports.

Technical Appendix Table 2. Number of cases of foodborne illness and number of cases with complete data for all study variables, by pathogen, Foodborne Diseases Active Surveillance Network (FoodNet) data, United States, 2004–2011

	No. cases (no. cases with complete data)		
Pathogen	Outbreak-associated cases	Sporadic cases	Outbreak fraction of all cases (cases with complete data), %
Campylobacter	201 (195)	47,887 (42,744)	0.4 (0.5)
Escherichia coli O157	736 (730)	3,165 (3,117)	18.9 (19.0)
Listeria	56 (56)	1,028 (1,024)	5.2 (5.2)
Salmonella	3,273 (3,161)	53,810 (50,690)	5.7 (5.9)

*Representing 110,157 total reports.

Technical Appendix Table 3. Percentage of all reported foodborne illness cases that were identified as outbreak-associated cases, by pathogen and selected characteristics, Foodborne Diseases Active Surveillance Network (FoodNet) data, United States, 2004–2011

	% Outbreak-associated cases			
Characteristic	*Campylobacter*	*Escherichia coli* O157	*Listeria*	*Salmonella*
FoodNet site				
California	0.0	1.5	1.7	3.0
Colorado	1.0	38.9	32.9	8.5
Connecticut	0.1	17.0	0.0	6.3
Georgia	0.2	8.4	0.0	2.6
Maryland	0.5	13.8	0.7	4.1
Minnesota	0.5	20.1	3.4	10.2
New Mexico	0.8	10.8	34.9	9.1
New York	0.4	22.8	3.7	8.2
Oregon	0.8	25.5	8.1	20.4
Tennessee	0.4	11.9	0.0	3.1
Year				
2004	0.1	9.0	0.8	5.4
2005	0.7	22.6	1.5	4.6
2006	0.6	15.9	4.3	7.1
2007	0.1	17.7	0.0	6.1
2008	0.6	25.7	0.0	7.8
2009	0.3	26.2	0.0	5.4
2010	0.4	21.1	2.3	5.1
2011	0.5	11.7	30.3	4.5
Age quintile				
1	0.6	20.5	2.3	2.1
2	0.6	18.1	4.6	4.2
3	0.3	19.2	5.1	9.0
4	0.3	19.3	5.5	7.5
5	0.3	17.2	8.3	5.8
Season quintile				
1	0.4	18.5	2.3	6.9
2	0.4	19.7	0.9	7.4
3	0.6	18.6	4.1	5.6
4	0.6	20.3	16.1	4.2
5	0.1	17.3	2.3	4.5
Sex				
F	0.4	19.4	6.4	6.1
M	0.4	18.4	3.8	5.4
Hospitalization				
No	0.5	20.1	4.1	6.3
Yes	0.3	17.5	5.3	4.8

*Age of persons with cases and season of specimen submission are classified by quintile of reported age and quintile of the day of year of the specimen submission date.
